# Viral Suppression of RIPK1-Mediated Signaling

**DOI:** 10.1128/mBio.01723-21

**Published:** 2021-08-10

**Authors:** Darshika J. Udawatte, Alan L. Rothman

**Affiliations:** a Institute for Immunology and Informatics, University of Rhode Island, Providence, Rhode Island, USA; b Department of Cell and Molecular Biology, University of Rhode Island, Providence, Rhode Island, USA; Istituto Pasteur-Fondazione Cenci Bolognetti; Albert Einstein College of Medicine

**Keywords:** apoptosis, inflammation, innate immunity, necroptosis, receptor-interacting protein kinase, signal transduction, virus-host interactions

## Abstract

Receptor-interacting serine/threonine-protein kinase 1 (RIPK1) has emerged as a key upstream regulator of cell death and inflammation. RIPK1-mediated signaling governs the outcome of signaling pathways initiated by tumor necrosis factor receptor 1 (TNFR1), Toll-like receptor 3 (TLR3), TLR4, retinoic acid-inducible gene 1 (RIG-I)/melanoma differentiation-associated protein 5 (MDA-5), and Z-binding protein 1 (ZBP1) by signaling for NF-κB activation, mitogen-associated protein kinase (MAPK) and interferon regulatory factor 3/7 (IRF3/7) phosphorylation, and cell death via apoptosis and necroptosis. Both cell death and inflammatory responses play a major role in controlling virus infections. Therefore, viruses have evolved multifaceted mechanisms to exploit host immune responses by targeting RIPK1. This review focuses on the current understanding of RIPK1-mediated inflammatory and cell death pathways and multiple mechanisms by which viruses manipulate these pathways by targeting RIPK1. We also discuss gaps in our knowledge regarding RIPK1-mediated signaling pathways and highlight potential avenues for future research.

## INTRODUCTION

Receptor-interacting serine/threonine-protein kinase 1 (RIPK1) was first discovered as a protein that interacts with the death receptor, Fas (1), in the signal transduction pathway leading to programmed cell death (2, 3). Since then, RIPK1 has emerged as a central molecule regulating multiple biological pathways leading to cell death and inflammation. Its role is of particular interest in immune regulation. The primary goal of the immune system is to protect the host from invading pathogens. For this purpose, the innate immune system has evolved multiple signaling networks that culminate in the production of cytokines, chemokines, and interferons. RIPK1 is located at the intersection of several of these signaling pathways and mediates signaling from both membrane-bound and intracellular receptors that are important in pathogen recognition. Consequently, pathogens, including viruses, target RIPK1 to evade host immune responses and thereby to establish effective infections. There is an unexpected diversity in the strategies exploited by viruses to target RIPK1. This review summarizes the current knowledge of RIPK1 signaling in innate immune regulation and the various mechanisms utilized by viruses to inhibit RIPK1 signaling, with the goal of understanding how these effects benefit viruses.

## THE STRUCTURE OF RIPK1

**FIG 1:**
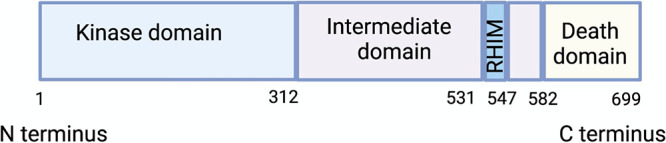
Domain structure of RIPK1. RIPK1 consists of an N-terminal kinase domain, an intermediate domain, and a C-terminal death domain.

**FIG 2:**
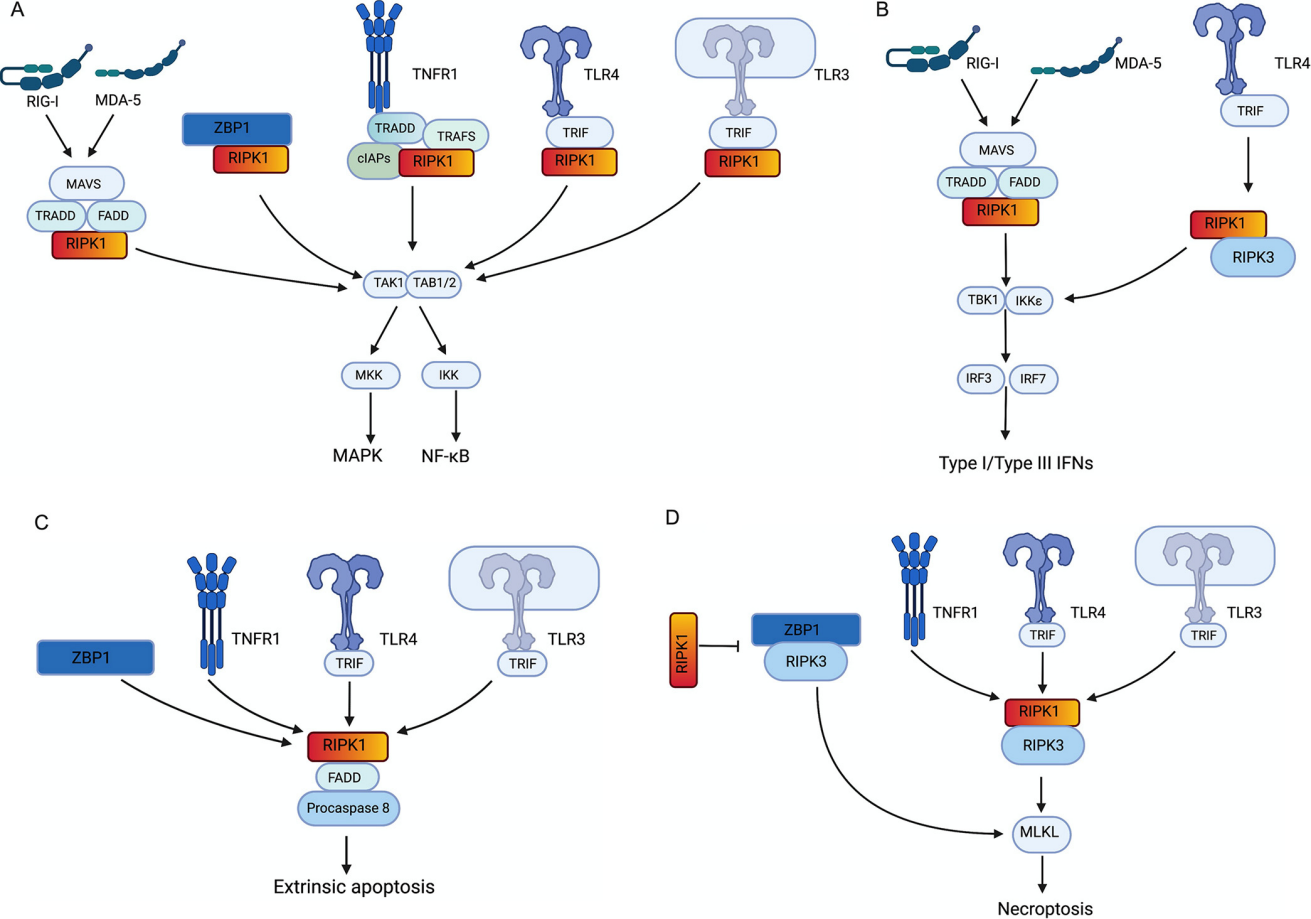
Signaling pathways known to be mediated by RIPK1. (A) RIG-I/MDA-5, ZBP1, TNFR1, TLR4, and TLR3 signal for assembly of TAK1 and TAB1/2 via RIPK1, which leads to NF-κB activation or MAPK signaling. (B) RIG-I/MDA-5 and TLR4 drive IRF3/IRF7-driven IFN production via RIPK1. (C) ZBP1, TNFR1, TLR4, and TLR3 signal for assembly of a complex consisting of RIPK1, FADD, and procaspase 8. This assembly results in maturation of procaspase 8 and initiation of extrinsic apoptosis. (D) Stimulation of TNFR1, TLR4, or TLR3 leads to interaction between RIPK1 and RIPK3, which results in the induction of necroptosis. ZBP1-RIPK3-driven necroptosis is antagonized by competitive binding of RIPK1 to ZBP1. Additional signaling pathways for these receptors that have not yet been proposed to involve RIPK1 are not shown.

RIPK1 is a member of the receptor-interacting serine/threonine family. RIPK1 harbors an N-terminal kinase domain, a RIP homotypic interaction motif (RHIM)-containing intermediate domain (ID), and a C-terminal death domain (DD) (1, 4, 5) (Fig. 1). The kinase activity and the ability of RIPK1 to interact with other DD-containing and RHIM-containing proteins make it a multifunctional protein involved in many cellular pathways (6, 7). RIPK1 mediates signaling downstream of tumor necrosis factor receptor 1 (TNFR1), Toll-like receptor 3 (TLR3), TLR4, retinoic acid-inducible gene 1 (RIG-I)/melanoma differentiation-associated protein 5 (MDA-5), and Z-binding protein 1 (ZBP1) (Fig. 2). These signaling events culminate in NF-κB-, mitogen-associated protein kinase (MAPK)-, and IRF3/7-mediated cytokine, chemokine, and interferon production, which is vital for controlling infections. Furthermore, RIPK1 signals for cell death mechanisms, including apoptosis and necroptosis.

## RIPK1 SIGNALING DOWNSTREAM OF TNFR1

Tumor necrosis factor alpha (TNF-α) ligation with TNF receptor 1 (TNFR1) initiates signaling cascades leading to NF-κB activation, MAPK activation, extrinsic apoptosis, and necroptosis (Fig. 2). RIPK1 acts as a critical signal transducer in the NF-κB pathway downstream of TNFR1. NF-κB signaling is vital for the regulation of the immune system. NF-κB family members (p50, p52, RelA, c-Rel, and RelB) regulate the transcription of proinflammatory cytokines and antimicrobial effectors such as interleukin-6 (IL-6), IL-8, IL-10, cc chemokine ligand 5 (CCL5), TNF-α, and cc chemokine receptor type 5 (CCR5) (8). They also regulate genes involved in cellular proliferation, differentiation, and survival (9–12). Pathogens have evolved mechanisms to downregulate NF-κB activation and evade these host responses (13). For example, human rotavirus and dengue virus (DENV) antagonize the NF-κB pathway, leading to downregulated cytokine responses (14, 15).

The role of RIPK1 in TNF-α signaling is well studied. Upon TNF-α ligation, TNFR1-associated death domain protein (TRADD) binds to the cytoplasmic death domain (DD) of TNF receptor 1 (TNFR1). TRADD recruits TNF-associated factors (TRAF2/TRAF5) along with RIPK1 and cIAP (cellular inhibitor of apoptosis) proteins. Ubiquitination of RIPK1 by TRAF2 and/or cIAPs creates a platform for recruiting transforming growth factor β-activated kinase 1 (TAK1), TAK binding proteins (TAB1 and TAB2), and the IκB kinase (IKK) complex (NF-κB essential modulator [NEMO], IKKα, IKKβ) (16–18). This protein complex is known as complex I. The IKK complex phosphorylates IκBα and promotes its proteasome-mediated degradation. This liberates NF-κB from the inhibitor IκBα and allows NF-κB to translocate into the nucleus, resulting in induction of antiviral genes. Studies done on mice with a genetic mutation in RIPK1 destroying its kinase activity demonstrated intact NF-κB responses following TNF-α stimulation. Furthermore, treatment with necrostatin-1 (Nec-1), a specific inhibitor of RIPK1 kinase activity, did not affect TNF-α-induced NF-κB responses in mice (19, 20). These data indicate that the kinase activity of RIPK1 is dispensable for NF-κB activation. Therefore, the scaffolding function of RIPK1 may play a major role in driving signals upstream of NF-κB. However, there is no conclusive evidence of the role of the RHIM domain of RIPK1 in protein-protein interactions in complex I.

MAPKs (mitogen-associated protein kinases) consist of three groups, Erk1/2 (extracellular signal-regulated kinases), JNKs (Jun N-terminal kinases), and p38 (21, 22). The MAPK pathway culminates in AP-1 transcription and induces the expression of genes important for host defense. AP-1 regulates the expression of proinflammatory cytokines and chemokines such as IL-2, interferon gamma (IFN-γ), TNF-α, IL-6, IL-4, granulocyte-macrophage colony-stimulating factor (GM-CSF), and C-X-C motif ligand 8 (CXCL8). Thereby, AP-1 regulates leukocyte activation and differentiation (23, 24). Numerous pathogens target the MAPK pathway to evade these host responses. For example, DENV did not activate Erk1/2 phosphorylation and even impaired the basal level of p-Erk1/2 in A549 cells (15). Herpes simplex virus 1 (HSV-1) protein Us3 suppressed Erk activity in HSV-1-infected cells (25).

RIPK1 is placed at a crucial point in MAPK signal transduction downstream of TNFR1. As noted above, RIPK1 ubiquitination in complex I recruits multiple additional proteins, including TAK1. TAK1 phosphorylates MAPKs such as p38, JNK, and Erk, leading to the expression of additional transcription factors, ATF2, JUN, and TCF, which translocate into the nucleus facilitating AP-1 transcription (26). One study found that the ID of RIPK1 was necessary and sufficient for the activation of MAPKs upon TNF-α stimulation (27). However, another study demonstrated (28) that RIPK1 interacted with MEKK3 (mitogen-activated protein kinase kinase kinase 3) via both the ID and the KD and mediated TNF-α-induced p38 MAPK activation by recruiting MEKK3 to TNFR1 (28). Additionally, a RIPK1-MEKK3 interaction was observed in cells expressing wild-type RIPK1 but not in cells expressing kinase-inactive RIPK1. These reports suggest that there may be more than one route of MAPK activation in complex I via RIPK1. Targeted disruption of the *rip1* gene (which codes for RIPK1) in mouse embryonic fibroblasts prevented p38 MAPK activation and IL-6 production in response to TNF-α (28), demonstrating the vital role of RIPK1 in regulating MAPK-AP-1 signaling downstream of TNFR1.

## RIPK1 SIGNALING DOWNSTREAM OF TLR3 AND TLR4

Stimulation of toll like receptors (TLRs) can lead to potent innate immune responses via NF-κB activation. TLR4, the sensor for bacterial lipopolysaccharide (LPS), triggers NF-κB activation via either MyD88 (myeloid differentiation primary response gene 88)-dependent or TRIF (TIR domain-containing adapter inducing interferon β)-dependent signaling pathways (29, 30). TLR3 participates in recognition of pathogen-associated double-stranded RNA (dsRNA) and the synthetic analog of viral dsRNA, poly(I·C). Unlike TLR4, TLR3 induces NF-κB activation only via TRIF (31). In both TLR3 and TLR4 signaling, TRIF interacts with RIPK1 via a conserved four-amino acid motif (VQLG) in the C-terminal RHIM domain of TRIF (32). TRIF also recruits TRAF6 and TBK1 via its N terminus (31). These interactions result in IKK activation, IκB phosphorylation, and NF-κB nuclear translocation (33). TRIF-RIPK1 interaction results in recruitment of TAK1 leading to the activation of MAPKs and AP-1 activation leading to enhanced immune responses (34). Even though this tethering of signaling molecules by RIPK1 (scaffolding function) is sufficient for NF-κB and MAPK activation, a RIPK1 kinase activity-dependent mechanism was recently reported (35). The kinase activities of RIPK1 and RIPK3 promoted sustained activation of Erk, cFos, and NF-κB in LPS-stimulated macrophages, resulting in inflammatory gene expression. Even though the speculation is that these inflammatory responses initiated from a death signaling complex involving RIPK1, referred to as the necrosome, the detailed molecular mechanism remains to be elucidated. It is tempting to conjecture that similar RIPK1 kinase activity-dependent signaling could occur downstream of TLR3 as well.

RIPK3 has been shown to inhibit RIPK1-mediated NF-κB activation by competitive binding to TRIF downstream of TLR3 and TLR4 (31). This RIPK3-TRIF interaction could potentially inhibit RIPK1-driven MAPK activation, although this has not been investigated so far. Therefore, it could be of interest to study the effect of RIPK3 on the TLR3/TLR4-MAPK pathway.

TLR3 and TLR4 activate signaling pathways through IRF3/IRF7 phosphorylation that culminate in the production of IFNs and IFN-inducible genes, which are central to antiviral innate immune responses (36). Even though RIPK1 is known to interact with TRIF, involvement of RIPK1 in TRIF-dependent IRF3/IRF7 signaling is less clear. Overexpression of the RHIM domain of RIPK1 alone was sufficient to induce TRIF-mediated NF-κB activation as measured using a luciferase reporter. However, the activity of an IFN-β luciferase reporter was not induced with overexpression of RIPK1 (31). Interferon-sensitive response element (ISRE) reporter luciferase activity increased in HEK293 cells with overexpression of TRIF but not with overexpression of RIPK1 (37). This RIPK1-independent response in IRF3/7 phosphorylation could be due to the ability of TRIF to directly interact with both IRF3 and the IRF3-activating kinase, TBK1 (33). However, another study demonstrated that a RIPK1-RIPK3 signaling complex mediated TRIF-dependent IFN-β synthesis in response to LPS in mice (38).

## RIPK1 SIGNALING DOWNSTREAM OF RIG-I AND MDA-5

The RNA helicases RIG-I (retinoic acid-inducible gene 1) and MDA-5 (melanoma differentiation-associated protein 5) are known to initiate signaling pathways culminating in the production of type I interferons (IFN-α, IFN-β, IFN-ε, IFN-κ) and proinflammatory cytokines during the early stages of viral infections (39). RIG-I most strongly recognizes short 5′-ppp dsRNA, while MDA-5 preferentially recognizes long dsRNA (40, 41). RIG-I most potently detects influenza viruses, filoviruses, and rhabdoviruses, while MDA-5 is essential for the recognition of picornaviruses, arteriviruses, and hepatitis D virus (42–46). These receptors are also involved in recognizing nonviral pathogens; MDA-5 has been reported to respond to malaria parasites (47). Nevertheless, viruses have evolved strategies to impair signaling downstream of RIG-I/MDA-5. For example, the DENV protease NS3 was shown to cleave mitochondrial-associated proteins MFN1 and MFN2, impairing efficient RLR (RIG-I like receptor) signaling (48). Influenza A H1N1 viral protein NS1 directly interacts with RIG-I and prevents its activation, inhibiting IFN-β production (49).

RIPK1 is involved in NF-κB, MAPK, IRF3, and IRF7 activation downstream of RIG-I and MDA-5. RIG-I and MDA-5 signaling merge at the mitochondria through MAVS (mitochondrial antiviral signaling protein), which is located at the mitochondrial membrane (50). TNFR1-associated death domain (TRADD) and Fas-associating protein with death domain (FADD) recruit RIPK1 to MAVS following RIG-I/MDA-5 stimulation (51). RIPK1 signaling to NF-κB proceeds through the IKK complex, while signaling to IRF3/IRF7 proceeds through TBK1 and IκB kinase-ε (IKKɛ) (52, 53). Activation of MAPK downstream of RIG-I involves TRAF2, TAK1, and the kinase activity of RIPK1 (54). RIG-I and MDA-5 activate NF-κB and p38 MAPK through a common upstream pathway involving RIPK1 and only diverge downstream of TAK1. Therefore, RIPK1 functions as an essential adaptor molecule in RIG-I and MDA-5 signal transduction by facilitating the association of other proteins (53). The kinase function of RIPK1 may not play a major role downstream of RIG-I/MDA-5. Evaluation of signaling downstream of RIG-I/MDA-5 with genetic or pharmacological inhibition of RIPK1 kinase activity would be needed to test this hypothesis.

## RIPK1 SIGNALING DOWNSTREAM OF ZBP1

ZBP1 (Z-binding protein 1), also known as DAI (DNA-dependent activator of IFN regulatory factors) or DLM1, was initially recognized as a sensor for double-stranded DNA (dsDNA) but later found its role as an RNA sensor during virus infections (55–58). Further studies revealed that ZBP1 can sense viral and endogenous Z-RNA (58, 59). ZBP1 acts as a vital innate immune sensor by regulating programmed cell death and inflammation during pathogen invasions. For example, ZBP1 has been shown to initiate signaling for cell death via either apoptosis, necroptosis, or pyroptosis during influenza A virus (IAV) infection (57, 60). ZBP1 regulates inflammatory responses by signaling for NF-κB activation and IRF3-driven IFN and cytokine production (7, 55, 61).

ZBP1 harbors three RHIM-like domains, and two of them have been shown to facilitate interactions with other RHIM domain-containing proteins (7). RIPK1 has been shown to mediate NF-κB activation by interacting with ZBP1 via its RHIM domains (7, 61). Consistent with this, secretion of IL-6 and TNF-α was suppressed in the supernatants of *RIPK1^−/−^* and *ZBP1^−/−^* cells relative to wild-type (WT) or *RIPK1^KD/KD^* bone marrow-derived macrophages (BMDM) infected with IAV (62). However, unlike in TRIF signaling, RIPK3 also contributes to NF-κB activation in ZBP1 signaling (7, 61). RIPK1 was detected as a critical component in relaying signals for IFN-β induction upon stimulation of ZBP1 with poly(dA:dT) (7).

Adding complexity to this system, an antiviral signaling mechanism involving ZBP1 and RIPK1 that is independent of cell death has also been reported. ZBP1 induced the expression of IRG1 via IRF1, and this was dependent on the expression of both RIPK1 and RIPK3. IRG1 acted as an antiviral effector, restricting Zika virus (ZIKV) replication in neurons by altering cellular metabolism. Mice expressing a kinase dead version of RIPK1 (*Ripk1^KD/KD^*) exhibited lower levels of *Irg1* mRNA and higher ZIKV titers in brain homogenates and mortality relative to wild-type controls (63). However, the precise mechanism, including the importance of RHIM interactions in relaying signaling in the ZBP1-RIPK1-IRG1 axis, is yet to be determined.

Overall, ZBP1-RIPK1 signaling has emerged as a crucial antiviral response by inducing NF-κB activation, proinflammatory cytokine secretion, and neuronal metabolic reprogramming. However, the importance of ZBP1-RIPK1 interactions in IFN induction via IRF3 remains unclear. Furthermore, ZBP1-RIPK1 signaling may exert a negative effect during viral infections by inhibiting RIPK3-dependent necroptosis. Future studies should address how RIPK1-ZBP1 interactions affect the scaffolding function of RIPK1 involving interactions with other RHIM-containing proteins such as TRIF and murine cytomegalovirus (MCMV) M45.

## RIPK1 SIGNALING IN CELL DEATH

### RIPK1-mediated apoptosis.

Viruses are obligate intracellular pathogens. Hence, cell death can be an important immune defense against infection. RIPK1 regulates cell death pathways known as “ripoptocide” involving both apoptosis and necroptosis (64). Apoptosis is a noninflammatory form of cell death having morphological changes such as cell shrinkage, chromatin condensation, and plasma membrane blebbing (65). Apoptosis can be induced by extrinsic or intrinsic stimuli. Extrinsic apoptosis is mediated by death receptors such as TNFR1, whereas intrinsic apoptosis is driven by cellular stresses such as DNA damage, growth factor withdrawal, or endoplasmic reticulum (ER) stress leading to mitochondrial outer membrane permeabilization (65, 66). RIPK1 is involved in regulating extrinsic apoptosis downstream of TNFR1. Deubiquitination of RIPK1 leads to the formation of a complex with procaspase 8 and FADD in response to TNF-α stimulation, which is referred to as complex IIa (Fig. 1B) (67). Assembly of complex IIa leads to auto-processing and maturation of procaspase 8 into caspase 8, which initiates extrinsic apoptosis. A similar complex known as the ripoptosome assembles in response to TLR3 and TLR4 stimulation (68, 69). The core of the ripoptosome consists of RIPK1, FADD, and caspase 8 (69). The ripoptosome can also recruit additional proteins such as cFLIP (FLICE-like inhibitory protein) and RIPK3 depending on the cell type and stimulus (69). ZBP1 sensing of IAV RNA led to RIPK1-mediated apoptosis, suggesting a similar complex formation downstream of ZBP1 (57). As in complex IIa, the ripoptosome promotes caspase 8 activation. Mature caspase 8, in turn, activates downstream caspases such as caspase 3, resulting in apoptosis. Active caspase 8 also cleaves RIPK1 downstream of residue Asp324 in the ID. The C-terminal 38-kDa product generated from this cleavage is known to facilitate interactions between FADD and TRADD, further contributing to apoptosis. The expression of RIPK1 mutants resistant to caspase 8 cleavage protected cells against TNF-α-induced apoptosis, demonstrating the proapoptotic nature of cleaved RIPK1 (70). The induction of apoptosis by RIPK1 contributes to virus clearance by eliminating infected cells but does not cause inflammation.

### RIPK1-mediated necroptosis.

Necroptosis is considered an antiviral defense mechanism because it can initiate a strong proinflammatory response. Necroptosis is characterized morphologically by swelling of organelles followed by plasma membrane rupture (71–73). Necroptosis protects the host both by limiting pathogen replication in infected cells and by promoting inflammatory responses that stimulate host adaptive immunity (74). Necroptotic cell death is driven by a protein complex (necrosome) that includes RIPK1 and RIPK3. Necrosomes are formed downstream of TNFR1, TLR3, and TLR4 in the absence of caspase 8 activity (67, 75). RIPK1 and RIPK3 interact with and phosphorylate each other in the necrosome complex. Activated/phosphorylated RIPK3 recruits and phosphorylates MLKL (mixed-lineage domain-like protein). Phosphorylated MLKL forms tetramers, translocates to the cell membrane, and further polymerizes to form amyloid-like structures (76), resulting in cell membrane permeabilization and cell lysis (77).

Furthermore, ZBP1 is also known to trigger virus-induced necroptosis by activating RIPK3 (56, 78). RIPK1 has been shown to block RHIM-mediated interactions between ZBP1 and RIPK3 by competitively binding to ZBP1, thereby counteracting ZBP1-mediated necroptosis (79). Mice expressing a mutation in the RHIM domain of RIPK1 died at birth due to ZBP1-RIPK3-dependent necroptosis, indicating that the RHIM of RIPK1 is critical for inhibiting ZBP1-RIPK3 dependent necroptosis during development (80). However, neither the precise mechanism of RIPK1 inhibition of RIPK3-ZBP1 interaction nor the trigger that initiates ZBP1 activation during development has been elucidated.

Even though RIPK1 participates in extrinsic apoptosis and necroptosis, the loss of RIPK1 also sensitizes cells to TNF-α-induced apoptosis (81, 82), perhaps due to NF-κB inhibition. Therefore, a goal of future studies should be to understand what factors regulate the final effects of RIPK1 leading to cell survival or cell death.

## VIRAL STRATEGIES IN MODULATING RIPK1 SIGNALING

RIPK1 is placed at a crucial point controlling cell death and inflammation, governing the outcome of signaling pathways initiated by TNFR1, TLR3, TLR4, RIG-I/MDA-5, and ZBP1 and leading to NF-κB activation, MAPK, and IRF3/7 phosphorylation. Activation of these pathways results in release of proinflammatory cytokines and interferons, setting an antiviral state, and in destruction of the replication niche of obligate intracellular pathogens via cell death. Therefore, RIPK1 is as an attractive target for inhibition by different virus families.

## HERPESVIRUSES

Members of the *Herpesviridae* family have double-stranded DNA genomes. They are categorized into alpha-, beta-, and gammaherpesviruses based on their host range, genome, and replication strategies (83). Herpesviruses commonly establish a lifelong infection in the host. Human herpes simplex virus-1 (HSV-1), an alphaherpesvirus, is associated with genital herpes, herpes encephalitis, and ocular HSV causing blindness (84). Human cytomegalovirus (CMV) is a betaherpesvirus that causes a wide spectrum of pathological outcomes. Congenital CMV infections are associated with serious complications, including microcephaly and mental retardation. Immunocompromised patients can develop encephalitis, pneumonitis, and graft rejection as a result of CMV infections (85). Epstein-Barr virus (EBV), a human gammaherpesvirus, is the primary cause of infectious mononucleosis. EBV infections are also associated with ∼200,000 malignancies (e.g., lymphoma, nasopharyngeal carcinoma, and gastric adenocarcinoma) worldwide every year (86). Several members of the herpesvirus family have been shown to impair RIPK1-mediated signaling.

### Herpes simplex virus-1 (HSV-1).

HSV-1 ICP6 protein induces RIPK3-mediated necroptosis in murine cells, which restricts viral propagation (87, 88). In contrast, HSV-1 has evolved to escape necroptosis-mediated restriction in human cells by at least three mechanisms. In one mechanism, the human HSV-1 protein ICP6 promotes RIPK1 aggregation and then its degradation by the autophagosome, a process known as aggrephagy (89). A conserved motif in all the human herpesviruses known as the “induced protein aggregation motif” (IPAM) is needed for this aggregate formation. Expression of wild-type ICP6 alone was sufficient to induce aggregate formation, while expression of a mutant IPAM (ΔIPAM) ICP6 failed to do so (90). However, no experiments were done to indicate if HSV-1 IPAM was important for RIPK1 interaction. In a second mechanism, it was shown that HSV-1 ICP6 and HSV-2 ICP10 both harbor RHIM domains and disrupt the RIPK1-RIPK3 association by competing for RIPK1 binding (91). This effect allows HSV-1 to prevent necrosome formation. Recently, an additional mechanism of necroptosis inhibition by HSV-1 ICP6 via the RHIM domain was discovered. Wild-type (WT) HSV-1 inhibited translocation of necrosomes to detergent-resistant membranes compared with HSV-1 lacking ICP6 (ΔICP6) or having a mutant ICP6 lacking the RHIM domain (ICP6-ΔRHIM) (92). Collectively, these data show numerous strategies exploited by HSV-1 to prolong cell survival by targeting RIPK1 in its natural host.

### Cytomegalovirus (CMV).

UL48 and UL45 of HCMV interacted with RIPK1 and suppressed RIPK1-mediated NF-κB activation. This was achieved by deubiquitylation of RIPK1 in complex I by UL48, which was enhanced by the expression of UL45 (93). The infection of mice with murine cytomegalovirus (MCMV) is a well-established model for understanding human CMV (HCMV) infections (94). MCMV M45, the homolog of HCMV UL45, has emerged as a viral inhibitor of RIPK1-mediated signaling. Mutational studies have revealed a RHIM-like domain in the N terminus of MCMV M45 (amino acids [aa] 61 to 69). Overexpressed WT but not ΔRHIM M45 coimmunoprecipitated with RIPK1, indicating that M45 interacts with RIPK1 via its RHIM domain (95). The interaction between RIPK1 and M45 resulted in blocking of RIPK1 ubiquitination (19). TNF-α-induced NF-κB activation and TNF-α-induced p38 phosphorylation were inhibited as a result of the RIPK1-M45 interaction (96). NF-κB activation downstream of both TLR3 and ZBP1 has also been shown to be blocked by M45 (61, 96). Furthermore, M45 blocked caspase-independent cell death following TNF-α stimulation in a RHIM-dependent manner. Similarly, MCMV expressing WT M45 but not ΔRHIM M45 blocked TRIF-induced apoptosis by blocking the RIPK1-TRIF interaction (95). Interestingly, another study found an additional mechanism of RIPK1 degradation by M45; M45 physically interacted with RIPK1 via its IPAM motif and caused RIPK1 to aggregate (90). RIPK1 levels were higher in autophagy-deficient *Atg5*^−/−^ cells following MCMV infection, indicating that RIPK1 was subjected to degradation by autophagy. M45 promotion of RIPK1 degradation was blocked by inhibition of lysosome function by NH_4_Cl (90). In agreement with previous studies, the authors also detected inhibition of necroptosis due to low levels of RIPK1. Both IPAM and RHIM sequences were needed for cell survival, suggesting that these two sequences have independent roles. Infections with MCMV expressing ΔIPAM or ΔRHIM M45 resulted in less viral replication in mice relative to infections with WT MCMV. Therefore, M45 promoted viral replication *in vitro* and *in vivo* by inhibiting RIPK1-mediated cell death.

### Epstein-Barr virus (EBV).

EBV is known to manipulate the host cell’s death signaling pathways to facilitate viral replication. EBV suppressed necroptosis by targeting RIPK1 (97). EBV latent membrane protein 1 (LMP1) directly interacted with RIPK1 in an RHIM-independent manner and increased both K48 and K63 ubiquitination of RIPK1. K48 ubiquitination resulted in a decrease of the RIPK1 half-life. LMP1 increased the expression of TRAF2, which amplified RIPK1 K63 ubiquitination. Therefore, LMP1 inhibited TNFR1-stimulated necroptosis by redirecting RIPK1 signaling toward NF-κB activation by promoting K63 ubiquitination. However, LMP1 is also known to upregulate TNFAIP3/A20 (TNF-α-induced protein 3), which could deubiquitinate RIPK1 (98, 99). Further studies of the effect of low levels of RIPK1 and LMP1-RIPK1 interaction on EBV replication and cell death could help to clarify which of these effects plays a dominant role.

## FLAVIVIRUSES

Flaviviruses are single-stranded positive-sense RNA viruses with a genome size of ∼11 kb. Flaviviruses are mainly transmitted to vertebrate hosts by infected mosquitos or ticks and cause a range of disease manifestations including nonspecific febrile illness, fever with rash, viral hemorrhagic fever, and encephalitis. Mosquito-borne flaviviruses include yellow fever virus, dengue virus, Japanese encephalitis virus, West Nile virus (WNV), and Zika virus (ZIKV). Tick-borne viruses include tick-borne encephalitis virus, Powassan virus, louping ill virus, and Omsk hemorrhagic fever virus (100). WNV is an encephalitic virus of global concern (101). In humans, ∼20% of WNV infections cause rashes, arthralgia, and myalgia while <1% of infections cause cognitive and neurological impairments that can lead to death (102). ZIKV is an emerging flavivirus that is associated with fetal abnormalities and severe neurological injuries in adults (103, 104). Studies of WNV and ZIKV have exposed a protective role of RIPK1 for the host, while the role of RIPK1-mediated signaling remains to be elucidated for other flaviviruses.

### West Nile virus (WNV).

RIPK1 together with RIPK3 signals for chemokine expression and for recruitment of infiltrating antiviral leukocytes in the central nervous system during WNV infection. Mice expressing a kinase dead form of RIPK1 exhibited increased susceptibility to WNV infection (105). Even though the molecular mechanisms for this effect are not completely understood, these data indicate a protective role of RIPK1 against WNV (105). Studies of the levels of RIPK1 upon WNV infection could help determine if WNV impedes RIPK1-mediated signaling.

### Zika virus (ZIKV).

The fate of RIPK1 in ZIKV-infected cells has not been directly addressed. However, a protective role of RIPK1 against ZIKV was recently reported. RIPK1 together with RIPK3 diminished ZIKV infection by activating antiviral gene networks, including IRF1-dependent Irg1 expression (63). The full nature of this mechanism remains to be elucidated. However, mice with a genetic defect in RIPK1 kinase activity (RIPK1^KD/KD^) had higher viral titers in whole-brain homogenates upon infection with ZIKV. Inhibition of kinase activity *in vivo* by treatment of wild-type mice with pharmacological inhibitors also increased brain viral burden. Furthermore, RIPK1^KD/KD^ neuronal cultures exhibited enhanced replication of ZIKV relative to wild-type controls. Interestingly, no differences were observed in ZIKV replication in primary BMDM or dendritic cells from wild-type versus RIPK1^KD/KD^ mice or with inhibition of the kinase activity of RIPK1, suggesting that these effects are cell-type specific (63).

## RETROVIRUSES

Retroviruses are enveloped RNA viruses and are entirely restricted to vertebrates (106). Retroviruses reverse transcribe their RNA genomes into DNA that integrates into the host cell genome as a critical step in the virus life cycle. It is estimated that approximately 8% of the human genome consists of endogenous retroviruses (107). Retroviruses are further categorized into lentiviruses, oncoretroviruses, and spumaviruses (108). Human immunodeficiency virus 1 (HIV-1) is a lentivirus. HIV-1 causes AIDS. Approximately 37 million people were living with HIV-1 worldwide as of 2017 (109). Human T-cell lymphotropic virus-1 (HTLV-1) is an oncoretrovirus. Infection of T-lymphocytes by HTLV-1 can cause adult T-cell leukemia (ATL) or HTLV-1-associated myelopathy (HAM) (110, 111). Both HIV-1 and HTLV-1 have been shown to modulate RIPK1 signaling.

### Human immunodeficiency virus 1 (HIV-1).

The HIV-1 protease (PR) plays an important role in the virus life cycle by cleaving viral polypeptides Gag and Gag-Pol into mature proteins (112). One study examined the effect of HIV-PR expression on RIPK1-mediated signaling (113). HIV-1 PR was shown to decrease both endogenous and overexpressed RIPK1. A catalytically inactive PR and the PR inhibitor saquinavir (SQV) prevented this cleavage. Mass spectroscopy studies revealed that PR cleaved RIPK1 at the ID domain. RIPK1 cleavage was confirmed in HIV-1-infected T cell lines and primary CD4^+^ T cells. PR cleavage of RIPK1 disrupted NF-κB activation as measured by luciferase reporter assays, and this activation was restored by the addition of SQV. Furthermore, PR overexpression impaired RIPK1-RIPK3 interaction as demonstrated by a yeast two-hybrid system. These data suggest that RIPK1 cleavage by PR can block necrosome formation inhibiting necroptosis. However, this study did not assess the effect of PR on RIPK1-mediated MAPK or IRF3/IRF7 activation. Collectively, these data indicate that HIV-1 PR can block multiple immune pathways by cleaving RIPK1. The effect of RIPK1 cleavage on HIV-1 replication remains to be studied.

### Human T-cell lymphotropic virus-1.

The HTLV-1-encoded Tax protein is a multifunctional protein that is crucial for T cell transformation by HTLV-1 (114). Tax inhibits innate immune signaling by multiple mechanisms involving RIPK1. Type I IFNs and interferon-stimulated genes (ISGs) are capable of inhibiting HTLV-1 replication. Therefore, viral evasion of IFN production and signaling probably represents a survival strategy. Overexpression of HTLV-1 Tax resulted in greater replication of vesicular stomatitis virus (VSV), an IFN-sensitive paramyxovirus, in primary mouse embryonic fibroblast (MEF) and Jurkat cells, demonstrating that Tax can inhibit host antiviral immune responses (115). HTLV-1-infected cells produced less IFN-β than uninfected cells upon stimulation with poly(I·C). Yeast two-hybrid screens and mutational constructs revealed a Tax interaction with RIPK1 via the RHIM in the ID domain of RIPK1 (115). However, the authors did not test for the presence of a RHIM domain in Tax protein. The Tax-RIPK1 interaction prevented the RIPK1-IRF7 interaction and inhibited IRF7 phosphorylation, downregulating the production of IFN-α and IFN-β. In contrast, Tax did not prevent RIPK1-RIG-I/MDA-5 interactions, and NF-κB signaling was unaffected as shown by luciferase assays (115). HTLV-1 is known to induce persistent activation of NF-κB, which contributes to T cell transformation (116). It was shown that Tax prevented TLR3-induced IRF3 phosphorylation but not NF-κB signaling by competitively binding to TRIF. However, this observation is inconsistent, since TRIF is located upstream of both IRF3 and NF-κB pathways. Therefore, it is reasonable to hypothesize that Tax might be inhibiting IRF3 phosphorylation at an additional step in TLR3 signaling.

## OTHER VIRUSES

### Human rhinovirus (HRV).

HRV is the major causative agent of the common cold and asthma exacerbations (117). There are approximately 170 serologically distinct HRV types in circulation (118). HRV is a single-stranded positive-sense RNA virus. The HRV genome encodes three proteases (2A, 3CD, and 3C), which are important for viral polyprotein maturation (119). These proteases have also been shown to cleave host proteins, including RIPK1. The HRV16 3C protease restricted host cell apoptosis and prolonged cell survival by cleaving RIPK1 in HeLa cells (120). The addition of a 3C protease inhibitor, rupintrivir, decreased RIPK1 cleavage. 3C-mediated RIPK1 cleavage was increased upon caspase 8 activation by actinomycin D (ActoD) relative to untreated cells. However, this observation of increased RIPK1 cleavage with activation of caspase 8 was not reproduced in HRV16-infected cells. Even though apoptosis induced with ActoD treatment decreased upon HRV16 infection, no evidence was shown that this was related to RIPK1 cleavage. Additional studies may be required for elucidating the direct effect of RIPK1 cleavage on cell death as well as HRV16 replication. Finally, given that full-length RIPK1 is decreased during HRV16 infection, it would be interesting to learn if RIPK1-mediated innate immune signaling such as NF-κB and MAPK pathways are disrupted during HRV infections.

### Ebola virus (EBOV).

EBOV is a negative-strand RNA virus belonging to the *Filoviridae* family (121). Ebola virus causes a viral hemorrhagic fever with a high fatality rate. Damage to host immune responses due to robust EBOV replication is considered to contribute to the disease severity. This excessive viral replication reflects the capability of EBOV to suppress innate immune mechanisms. For example, EBOV effectively blocks IFN production (122, 123). EBOV has been shown to disrupt RIPK1-mediated signaling by encoding an miRNA (miR-T2-3p) targeting RIPK1 (124). *In vitro* studies in HeLa cells demonstrated that a synthetic mimic of miR-T2-3p downregulated RIPK1 transcript levels. In light of this observation, it is tempting to hypothesize that the EBOV-encoded miRNA blocks RIPK1-mediated innate immune responses to enhance viral replication. RIPK1 inhibition could contribute to inhibition of IFN production by EBOV. Thus, it will be of interest to study the link between RIPK1 downregulation and IFN inhibition during EBOV infection. Also, the effect of RIPK1 inhibition on EBOV replication could be further explored.

### Vaccinia virus (VACV).

VACV belongs to the poxvirus family. It has a large linear dsDNA genome. VACV is the primary component of the smallpox vaccine but is mostly used as a tool in the research setting (125). RIPK1 has been shown to play a protective role in VACV infection by inducing necroptosis. Caspase 8 inhibition by VACV also promotes necroptosis in infected cells (126). Mice expressing a kinase-inactive RIPK1 mutant failed to control VACV replication *in vivo*. They had increased viral titers in the spleen and the liver due to lack of necroptosis (127). This study provides genetic evidence that the kinase activity of RIPK1 is essential for VACV-initiated necroptosis. However, further efforts are needed to understand RIPK1-mediated NF-κB, MAPK activation, and cytokine production during VACV infection.

## CONCLUDING REMARKS

As discussed in this review, RIPK1 plays a crucial role in cell death and inflammatory responses. RIPK1 is placed at an intersection of multiple pathways of signal transduction initiated from death receptors and pattern recognition receptors. Signaling initiated from these receptors is important for the induction of immune responses. Importantly, new properties of RIPK1 signaling are emerging. For example, it has long been postulated that the kinase activity of RIPK1 is dispensable for NF-κB activation (128). Nevertheless, a RIPK1 kinase activity-dependent mechanism of NF-κB activation and induction of inflammatory responses due to TLR4 stimulation was recently reported (35). RIPK1 is involved in driving signaling for extrinsic apoptosis in response to TNF-α stimulation. However, RIPK1 was also reported to be implicated in protecting hepatocytes from TNF-α-stimulated cell death, suggesting opposing roles of RIPK1 in the same pathway. Even though RIPK1 is involved in inducing necroptosis, it was shown to block ZBP1-mediated necroptosis (80). Furthermore, RIPK1 was shown to induce antiviral gene networks by undefined mechanisms (63, 105). These findings suggest that there is substantially more to be learned about the consequences of viral targeting of RIPK1.

Evidence demonstrating that RIPK1 is involved in suppressing cell death and inflammation while also promoting the same supports RIPK1 as a critical regulator in cell death and inflammation signaling networks. Under normal conditions, RIPK1 regulation of cell death and inflammation seems to be tightly controlled to allow homeostasis. However, in the setting of viral infection, this homeostasis appears to be disturbed. In most viral infections, RIPK1-mediated signaling protects the host by inducing either cell death or secretion of inflammatory cytokines and IFN, as illustrated by the studies of WNV, ZIKV, and VACV infection of RIPK1 kinase activity-deficient mice (63, 127). If the overall effect of RIPK1 is to inhibit infection, RIPK1 becomes an attractive target for inhibition by viruses, especially due to the fact that many signaling pathways can be blocked simultaneously. It is surprising yet fascinating to observe the wide spectrum of mechanisms utilized by viruses to inhibit RIPK1. As shown in Table 1, numerous viral proteins are implicated in inhibiting RIPK1-mediated signaling, and new virulence factors are emerging. For example, Nsp-12 of SARS-Cov-2 (severe acute respiratory syndrome coronavirus-2) was shown to interact with RIPK1, yet the implications of this interaction have yet to be explored (129).

**TABLE 1:** List of virulence factors and mechanisms that manipulate RIPK1 signaling

Virus	Affected RIPK1 signaling	Virulent factor	Mechanism	Reference(s)
HSV-1	Necroptosis	ICP6	RIPK1 aggregation and consequent degradation by the autophagosome Blocks RIPK1 signaling and translocation of necrosome components to the DRM vesicles Disrupts RIPK1-RIPK3 interactions	90 – 92
HSV-2	Necroptosis	ICP10	Disrupts RIPK1-RIPK3 interactions	91
HCMV	NF-κB activation	UL48, UL45	Deubiquitylation of RIPK1	93
MCMV	NF-κB activation, p38 phosphorylation Extrinsic apoptosis, necroptosis	M45	M45-RIPK1 interaction RIPK1 aggregation and consequent degradation by the autophagosome	61, 90, 95, 96
EBV	Necroptosis	LMP1	LMP1 interacts and ubiquitinates RIPK1	97
HIV-1	NF-κB activation Necroptosis	PR	Cleavage of RIPK1	113
HTLV-1	Type I IFN production	Tax	Tax-RIPK1 interaction	115
HRV16		3C protease	Cleavage of RIPK1	120
EBOV		miRNA (miR-T2-3p)	Inhibition of RIPK1 transcription	123

Understanding the viral strategies used for evasion of RIPK1 signaling has revealed additional multifunctionality of viral proteins. This is exemplified by the identification of RHIM and IPAM domains in viral proteins. The IPAM motif is present in more than 70 viral R1 homologs of herpesviruses and baculoviruses, suggesting that its function of inducing protein aggregation might be widely conserved (90). Understanding viral strategies of RIPK1 inhibition could potentially point toward unexplored viral-host interactions. Seeing such a variety of strategies evolved in viruses indicates the possibility that RIPK1 inhibition is more important for viral survival than has been known.

Since RIPK1 controls the outcome of many signaling pathways, the sum of the diverse functions attributable to RIPK1 may cooperatively contribute to the outcome. Therefore, it may be helpful to investigate the outcome of all the possible arms of innate immune responses controlled by RIPK1 during pathogen invasions, and especially how the outcome of RIPK1 signaling affects viral replication. Physiologic extracellular signals that initiate RIPK1-mediated signaling could differ between *in vivo* and *in vitro* infection. For example, extracellular pH, oxidants, and Ca^2+^ are known to trigger necroptosis (130). Therefore, the outcomes of RIPK1 inhibition in these settings could be different. For example, even though RIPK1 kinase-deficient mice exhibited high viral titers following ZIKV infections, similar results were not observed in BMDM s or dendritic cells (DCs) after infection with ZIKV (63).

How RIPK1 inhibition might benefit the virus over the host should also be more fully addressed. Necroptosis and inflammatory signaling regulated by RIPK1 may control viral replication but could be deleterious to the host and contribute to disease pathogenesis. For example, even though necroptosis following IAV infection acts as a very effective antiviral strategy, it is speculated to contribute to pulmonary tissue damage and acute respiratory distress syndrome (ARDS) (131, 132). At this point, however, there is still a limited understanding of the specific effects of RIPK1 inhibition in disease pathogenesis during viral infections.

In this review, we have discussed various strategies exploited by viruses to inhibit RIPK1-mediated signaling. Viral modulation of RIPK1-mediated signaling may inhibit NF-κB responses, MAPK signaling, IFN induction, and cell death. It appears from the literature cited above that most viruses benefit by inhibiting these pathways. Understanding the impact of inhibition of RIPK1 signaling on the host and the virus may facilitate the development of new immunotherapeutic strategies against virus infections.
